# Stochastic Frontier Approach and Data Envelopment Analysis to Total Factor Productivity and Efficiency Measurement of Bangladeshi Rice

**DOI:** 10.1371/journal.pone.0046081

**Published:** 2012-10-15

**Authors:** Md. Kamrul Hossain, Anton Abdulbasah Kamil, Md. Azizul Baten, Adli Mustafa

**Affiliations:** 1 Mathematics Section, School of Distance Education, Universiti Sains Malaysia, Penang, Malaysia; 2 Department of Decision Science, School of Quantitative Sciences, Universiti Utara Malaysia, Sintok, Darul Aman, Kedah, Malaysia; 3 School of Mathematical Sciences, Universiti Sains Malaysia, Penang, Malaysia; Université de Nantes, France

## Abstract

The objective of this paper is to apply the Translog Stochastic Frontier production model (SFA) and Data Envelopment Analysis (DEA) to estimate efficiencies over time and the Total Factor Productivity (TFP) growth rate for Bangladeshi rice crops (Aus, Aman and Boro) throughout the most recent data available comprising the period 1989–2008. Results indicate that technical efficiency was observed as higher for Boro among the three types of rice, but the overall technical efficiency of rice production was found around 50%. Although positive changes exist in TFP for the sample analyzed, the average growth rate of TFP for rice production was estimated at almost the same levels for both Translog SFA with half normal distribution and DEA. Estimated TFP from SFA is forecasted with ARIMA (2, 0, 0) model. ARIMA (1, 0, 0) model is used to forecast TFP of Aman from DEA estimation.

## Introduction

Total factor productivity (TFP) is a widely used measure to calculate productivity. There are two kinds of methods to calculate TFP: a) Stochastic Frontier Analysis (SFA), which is parametric, and b) Data Envelopment Analysis (DEA), which is nonparametric. The two alternative approaches have different strengths and weaknesses. The main advantage of DEA is that it does not require any information more than input and output quantities. The efficiency is measured relative to the highest observed performance rather than an average [1]. However, a DEA-based estimate is sensitive to measurement errors or other noise in the data because DEA is deterministic and attributes all deviations from the frontier to inefficiencies. The strength of SFA is that it considers stochastic noise in data and also allows for the statistical testing of hypotheses concerning production structure and degree of inefficiency. Its main weaknesses are that it requires an explicit imposition of a particular parametric functional form representing the underlying technology and also an explicit distributional assumption for the inefficiency terms. The rationale for using two competing approaches is to countercheck whether results obtained by one can be confirmed by the other.

Agricultural development has recently returned to the forefront of development issues, drawing attention to the impacts of agricultural productivity change on economic growth and poverty reduction in both rural and urban areas. Agricultural productivity and its determinants are important but have not always been well measured or well understood. Bangladesh currently uses 75% of its arable land to produce 94% of country's total food grain requirement. Few researchers have done studies on the TFP growth rate of Bangladesh crop agriculture. Most studies consider the rice production as one crop, for example, Ahmed [2] examines the factor behinds the growth of TFP of rice and the market-orientated policy reforms, Alam et al [3] measured the TFP for the period of premarket reform (1987) and postmarket reform (2000 and 2004), Coelli et al [4] reported the TFP growth, technical efficiency change and technological change in Bangladesh crop agriculture for the 31 observations from 1960/1961 to 1991/1992, using data for 16 regions. Wadud and White [5] compared the efficiency of SFA and DEA of rice farm household in Bangladesh. They collected data from two villages by a survey conducted in August and September in 1997. Adachi *et al* [6] determined the factors' effects on productivity of Boro and Aman.

Productivity growth from new agricultural technology was declining, and that trend was a threat to sustainable economic development of Bangladesh in 1990s [7]. Although overall food production steadily increased, the yield of modern rice varieties declined from 3.6 tons per hectare in 1969 to 2.4 tons in 1994 [8]. The production of rice is not enough to feed the nation, and 1.5 million tons of annual shortage of food grain exists. Bangladesh will require about 27.26 million tons of rice for the year 2020. During this time, total rice area will also shrink to 10.28 million hectares. Therefore, rice yield needs to be increased from the present 2.74 to 3.74 tons per hectare. To gain full self-sufficiency in rice production, the rice production has to be increased by making use of the available technologies [9]. To solve the shortage, Bangladesh needs to know the condition of the total factor productivity of rice and efficiency by type of rice.

Measures of efficiency indicate the possibility of improvement in total productivity. Nguyen et al. [10] measured efficiency of rice farms in South Korea. They concluded that without major policy interventions, rice farms could improve economic and environmental performance by being more technically efficient. Alene [11] measured and compared TFP growth in African agriculture over the period 1970 to 2004. The principal source of growth was found as technical change, rather than as efficiency change. However, to our knowledge, no study on the comparative TFP growth rate and technical efficiency of the three types of rice (Aus, Aman and Boro) grown in Bangladesh has been carried out. The aim of this paper is to estimate the efficiency level and TFP as well as to develop model for forecasting the TFP of rice production in Bangladesh. For this purpose, we calculate the TFP of rice production: a Translog production function with different distribution assumptions for the inefficiency term and DEA model are estimated. The growth rate of total factor productivity estimates obtained from the two techniques is compared. Finally, ARIMA (p,d,q) model is used to forecast TFP. The novelty of this article is in using SFA and DEA model separately for Aus, Aman, and Boro to calculate the TFP of Bangladeshi rice and forecast the TFP from SFA and DEA through ARIMA model.

## Methodology

### 1. Parametric Stochastic Frontier Model

A stochastic frontier production model with time-varying inefficiency is used in panel data. Functional form of translog production model in SFA can be defined as
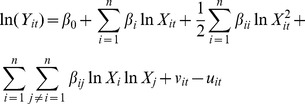
(1)where *Y_it_* = Output in the i- th firm in the t- th period

*X_it_* = input variables in the i- th firm in the t- th period

β_0_, β_i_, β_ii_ = the unknown parameter to be estimated.

The systematic error component, v_it_, is assumed to be independently and identically distributed random error having normal distribution with mean zero and variance σ_v_^2^, that is, N(0, σ_v_^2^). and u_it_ stands for technical inefficiency and can be predicted by the following equation:
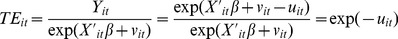
(2)u_it_ is measured as the ratio of observed output to the corresponding stochastic frontier output. It takes a value between zero and 1. u_it_ = (u_i_*exp(-ç(t-T))) where u_it_ is the non-negative random variables, which are assumed to account for technical inefficiency in production and are assumed to be independent and identically distributed as truncations at zero. It may be follow truncated normal, half normal, or exponential. ç is a parameter to be estimated, and the panel of data need not be complete (i.e., unbalanced panel data); t is the period of calculation inefficiency, whereas T is the end period.

Technically, we use the methodology to calculate TFP from [12]. The Malmquist index measures changes in the TFP between two observed points as a ratio of the distance functions of each point relative to a frontier production model, the efficiency change index for firm i is the ratio of the observed technical efficiency in time period t to that in time s, that is,
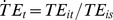
(3)The technical change index for firm i between period s and period t is computed as the geometric mean of two partial time derivations of the production function, that is,
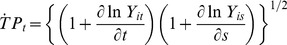
(4)The product of these two indices gives the Malmquist TFP index, showing the TFP change between period s and period t, that is,
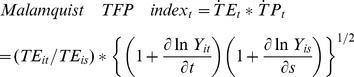
(5)


### 2. Empirical Stochastic Frontier Model

The functional form of translog stochastic frontier production model is defined as follows:
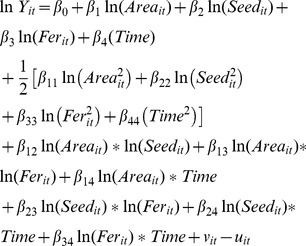
(6)where

i = 1, 2, 3 ; t = 1, 2, 3……..20

*Y_it_* = production in the i-th rice with t-th period.

*Area_it_* = area in the i-th rice with t-th period

*Seed_it_* = the quantity of seed of the -th rice in the i-th rice with t-th period

*Fer_it_* = the amount of fertilizer used in the i-th rice with t-th period

*Time* = the periods vary from 1 to 20.

ln = the natural logarithm

i = the number of rice group

### 3. Non-parametric DEA Model: Malmquist Index

The Malmquist Productivity Index (MPI), a nonparametric DEA model under time-dependent situations, is used for the evaluating the TFP growth of a Decision Making Unit (DMU). The concept of MPI that was introduced by Malmquist [13] further developed as the input-output distance function by Shephard [14] and forwarded by Fare and Grosskopf [15].

The Malmquist index in terms of the input and output data in t and t+1 time is denoted by sets (x^t^,y^t^) and (x^t+1^,y^t+1^) as
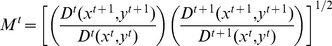
(7)where the distance function D^t^(x^t^, y^t^) = 1/F^t^(y^t^, x^t^|C,S) is the reciprocal of Farrel technical efficiency and the proportion of the production point (x,y), which is compressed to the ideal minimum input point. X_j_ = (x_1j_, x_2j_, x_3j,…_, x_mj_) and Y_j_ = (y_1j_, y_2j_, y_3j,…_, y_nj_) represent the input and output vector, respectively. F^t^(y^t^, x^t^|C,S) is the Farrel technical efficiency, and (C,S) is the input set on the production with input strong disposability.

The Malmquist index can be decomposed into both technical efficiency change (TEC) and the technical change (TC), which is as follows:
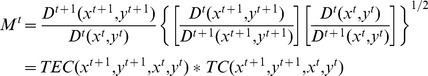
(8)


### 4. Autoregressive Integrated Moving Average (ARIMA) model

Sections 2.1 and 2.3 provide TFP according to parametric and nonparametric estimation, respectively. TFP is a univariate time series data. ARIMA (p,d,q) model can be used to forecast TFP where p is the order of autoregressive (AR) part, d is the number of difference to get stationary data, and q is the order of moving average (MA). ARIMA model building has the following steps:

STEP 1: Model Identification

Correlogram, the graph of Autocorrelation function (ACF) and Partial Autocorrelation function (PACF), is used to find the number of parameters for the ARIMA model.

STEP 2: Model Estimation

General form of ARIMA (p, d, q) can be expressed as
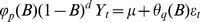
(9)where B is the backward linear operator or lag and BY_t_ = Y_t-1_.

Y_t_ is the time series data at time t, μ is the mean of time series data, ε_t_ is the error at time t, φ_p_ is the p-th parameter for AR, and θ_q_ is q-th the parameter for MA.

STEP 3: Model Checking

We have two types of information criterion: Bayesian Information Criterion (BIC) and Akaike Information Criterion (AIC). The best model has minimum value of AIC and BIC.
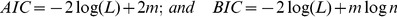
(10)where L is the likelihood of the model, n is the number of observation, and m ( = p+q) is the number of parameters in the model.

STEP 4: Forecasting with Model

Selected best model can be used for forecasting.

### 5. Data Sources and Types

The data on rice production in Bangladesh are obtained from The Year Book of Agricultural Statistics published by the Bangladesh Bureau of Statistics (BBS) every year. The Agriculture branch of BBS is responsible for collecting, compiling, and disseminating crop statistics and other agriculture-related data. Additionally, secondary data from other organizations are also included in the year book.

Information about the three types of rice are generally available, such Aus, Aman, and Boro in The Yearbook of Agriculture Statistics, Bangladesh. The dependent variable is production of rice, and independent variables are the area, amount of seed, and amount of fertilizer for each type of rice collected from Yearbook 2009. For this study, we consider 20-year period from 1989 to 2008. Because Bangladesh produces rice Aus, Aman, and Boro simultaneously, it is necessary to know the efficiency level of each type of rice separately, which motivates the use of the data set. To become efficient in rice production, it needs to improve the production of each of the three types of rice. To the best of our knowledge, it is the only existing comprehensive farm level data set available for the three types of Bangladeshi rice.

### 5.1 Output variable

#### Production

Among the three types of rice, Boro is the most important in Bangladesh because of its huge production. The favorable weather condition, electricity management, and stable market price helped the farmers to bring more area under the Boro crop in Bangladesh. Total Boro production has been estimated in thousand metric tons. Estimation of Boro production includes all the general varieties such as the local Boro and the hybrid Boro. Total production of Aus has been estimated in thousand metric tons. Varieties of Aus are local Aus and hybrid Aus, which are included in the data set. In case of Aman production, we include the amount of broadcast Aman, local transplant Aman, and HYV Aman in the data set.

### 5.2 Input Variables

#### Area

The total areas of Boro, Aus, and Aman rice have been estimated in thousand hectares. In this research, we considered using the land of all varieties of each crop as area.

#### Seed

Seed is a very important input to increase rice production. Therefore, it is very much required that the farmers must use pure, healthy seeds as per the minimum certification standards, which have standard percentage. In fact, the seed is the foundation of farming. Highly good-quality seeds are those that have genetic purity, physical purity, health standards, and moisture percentage in accordance with the minimum seed certification standards. If the seed is of bad or low quality/sub standard, then the labor and other expenses, which the farmer does, are in vain. For this study, we measured the amount of seed in thousand metric tons. Amount of improved seed for each crop is separately given in the Year Book of Agriculture.

#### Fertilizer

Fertilizer is kingpin in enhancing crop production. No country has been able to increase agricultural productivity without expanding the use of chemical industry. Fertilizer application mainly depends on soil type, growing season, irrigation applications, and cultivars used. Demand for fertilizer is also affected by agro climatic conditions. High yielding varieties of rice are highly responsive and need adequate supply of fertilizer to achieve targeted production. Urea (nitrogen), Muriate of Potash (MOP), and Triple Supper Phosphate (TSP) are the major fertilizers, which were applied in agricultural land in various proportions for rice production in Bangladesh. Type of fertilizer depends on the type of rice; for the limitation of methodology, we combine all the types of fertilizer quantity together with same unit (‘000 MT)

## Results and Discussion

Table 1 shows that the maximum-likelihood estimate of the parameters with time-varying inefficiency effects for area, seed, and fertilizer input is 0.429, 0.530, and −1.252 for half-normal distribution and 5.796, 2.552, and −4.968 for truncated normal distribution. For half normal, area and fertilizer are statistically significant at 1% and 5% level of significance, respectively. Seed is not a significant input in SFA with half-normal distribution, but all variables (area, seed, and fertilizer) are highly significant at 1% level of significance in SFA with truncated normal distribution. For both considering distribution in SFA, intercept is positive and significant. Therefore, intercept indicates that technological progress is improved over time.

**Table 1:** Maximum-Likelihood Estimates of the Translog Stochastic Frontier Model with Time-varying Inefficiency Effects for Rice production of Bangladesh.

Variable	Half normal			Truncated normal		
	Coefficient	std-error	t-ratio	Coefficient	std-error	t-ratio
Intercept 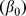	9.165*	13.279	1.690	39.226***	1.040	37.716
Area 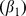	0.429***	2.393	−2.793	5.796***	0.751	−7.717
Seed 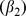	0.530	1.195	1.444	2.552***	0.952	2.6796
Fertilizer 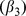	−1.252**	1.643	−2.062	−4.968***	0.357	−13.905
Time 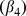	−0.1688	0.146	0.1580	−0.352***	0.135	−2.598
Area*Seed 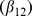	0.004**	0.004	1.960	0.005	0.008	0.6075
Area*Fertilizer 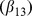	−0.002	0.003	−0.7489	−0.003	0.007	−0.3765
Seed*Fertilizer 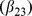	−0.004	0.003	−1.623	−0.005	0.004	−1.2795
Time*Area 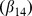	−0.0013	0.013	−0.0119	−0.035***	0.0127	−2.766
Time*Seed 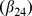	0.0000007	0.006	0.1202	0.0087	0.0077	1.124
Time*Fertilizer 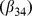	0.0036*	0.021	1.713	0.0732***	0.0151	4.863
Aera^2^ 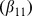	0.024	0.034	0.6996	0.028	0.0429	0.649
Seed^2^ 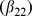	0.209*	0.3	1.6973	0.875***	0.120	7.278
Fertilizer^2^ 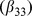	−0.0914*	0.133	−1.6866	−0.344***	0.095	−3.611
Time^2^ 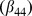	−0.0000003	0.001	−1.4481	−0.0013	0.001	−1.210
sigma-squared (σ^2^)	0.3025	0.243	1.244	0.0271***	0.005	5.485
gamma( 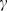 )	0.9963***	0.003099	321.46	0.9551***	0.017	55.757
Mu( 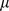 )	0	0	0	0.322***	0.093	3.469
Eta ( 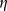 )	0.0146	0.01	1.456	0.0207***	0.006	3.292
	log likelihood function = 107.245			log likelihood function = 100.518		

***, ** and * indicates significance level at 1 percent, 5 percent and 10 percent respectively.

Inefficiency of time-varying production function is calculated by the error term. Composed error term of a stochastic frontier model is defined as γ = σ_u_^2^/(σ_u_^2^+σ_v_^2^) a measure of level of inefficiency. Range of inefficiency is between 0 and 1. Ratio of specific variability to total variability (γ) is positive and significant at 1% level of significance in both consideration (half-normal and truncated normal) of SFA, implying that specific technical efficiency is important in explaining the total variability of the output. In SFA with half-normal and truncated normal distribution, the value of γ is 0.996 and 0.955, respectively. That is, 99.6% and 95.5% of random variation occurred in production because of inefficiency measured by SFA with half-normal and truncated normal distribution, respectively. Estimated parameters of time-varying inefficiency model indicate that the technical inefficiency effects tend to decline over time because the estimate for the parameter eta (η) is positive in both consideration of SFA. Eta (η) is highly significant when considering inefficiency following truncated normal distribution but insignificant when considering half normal distribution in SFA. The value of mu (μ) is zero in SFA with half-normal, indicating that the distribution of the inefficiency effects is more concentrated than that of SFA with truncated normal. The second order coefficients (β_12_, β_34_, β_22_, β_33_) and (β_14_, β_34_, β_22_, β_33_) are significant in SFA with half normal and truncated normal distributions respectively.

Table 2 shows that average technical efficiency of rice production from 1989–1990 to 2008–2009 is 0.572 in SFA with truncated normal distribution, whereas the average efficiency is 0.639 in SFA with half-normal distribution. Results imply that 57.2% and 63.9% of rice production is being produced in Bangladesh according to the truncated normal distribution and half-normal distribution, respectively. That is why rice production in Bangladesh can improve 42.8% and 36.1%, respectively, by the same set of given inputs and technology. The half-normal distribution provides higher technical efficiency estimates than the truncated normal distribution. Overall average production of rice is only about 57.2% in SFA with truncated normal distribution, whereas it is about 63.9% with half-normal distribution.

**Table 2:** Year-wise Technical Efficiency of Rice in Bangladesh.

Year	Technical Efficiency (Half normal)				Technical Efficiency (Truncated normal)			
	Aus	Aman	Boro	Overall	Aus	Aman	Boro	Overall
1989	0.462	0.372	0.98	0.605	0.4	0.304	0.845	0.516
1990	0.467	0.378	0.981	0.608	0.407	0.311	0.848	0.522
1991	0.472	0.383	0.981	0.612	0.415	0.319	0.851	0.528
1992	0.477	0.388	0.981	0.616	0.422	0.326	0.854	0.534
1993	0.482	0.394	0.981	0.619	0.43	0.334	0.857	0.540
1994	0.488	0.399	0.982	0.623	0.437	0.341	0.86	0.546
1995	0.493	0.405	0.982	0.626	0.445	0.349	0.862	0.552
1996	0.498	0.41	0.982	0.63	0.452	0.357	0.865	0.558
1997	0.503	0.415	0.982	0.633	0.46	0.364	0.867	0.564
1998	0.508	0.42	0.983	0.637	0.467	0.372	0.87	0.570
1999	0.513	0.426	0.983	0.641	0.475	0.38	0.872	0.575
2000	0.518	0.431	0.983	0.644	0.482	0.387	0.875	0.581
2001	0.523	0.436	0.983	0.648	0.489	0.395	0.877	0.587
2002	0.528	0.442	0.984	0.651	0.496	0.402	0.88	0.593
2003	0.533	0.447	0.984	0.654	0.504	0.41	0.882	0.598
2004	0.537	0.452	0.984	0.658	0.511	0.418	0.884	0.604
2005	0.542	0.457	0.984	0.661	0.518	0.425	0.886	0.610
2006	0.547	0.463	0.985	0.665	0.525	0.433	0.889	0.615
2007	0.552	0.468	0.985	0.668	0.532	0.44	0.891	0.621
2008	0.557	0.473	0.985	0.672	0.539	0.448	0.893	0.626
Average	0.51	0.423	0.983	0.639	0.470	0.376	.870	0.572

It is clear from the annual technical efficiency of rice that the efficiency is increasing over time. Technical efficiency of Aus is increased from 0.462 in 1989 to 0.557 in 2008 and from 0.40 in 1989 to 0.539 in 2008 for half-normal and truncated normal distributions, respectively. The efficiency of Aman is increased from 0.372 to 0.473 and 0.304 to 0.448 for half normal and truncated normal distribution, respectively, and that of Boro is almost constant for both half-normal (0.98) and truncated normal (0.85) distributions.

There is wide variation in technical efficiencies among the different types of rice. Average efficiencies over time are 0.51, 0.423, and 0.983 for Aus, Aman, and Boro, respectively, from SFA with half normal. These values indicate that Aus, Aman, and Boro can improve their output level more by 49%, 57.7%, and 1.7% from the current level, respectively, using the same inputs and technology. Average technical efficiencies are found to be 0.47, 0.376, and 0.870 for Aus, Aman, and Boro, respectively, from SFA with truncated normal. These values indicate that Aus, Aman, and Boro can improve their output level more by 53%, 62.4%, and 13% from the current level, respectively, using the same inputs and technology. In both cases, half-normal and truncated normal of SFA, the lowest technical efficiency is observed for Aus production, and the highest technical efficiency is recorded for Boro production. The result is supported by [16]. They mentioned that the rank of efficiency does not change because of assumption of inefficiency distribution. Average overall technical efficiency is larger for SFA with half-normal distribution (0.639) than SFA with truncated normal distribution (0.572), which is contradictory with [17].

Table 3 compares SFA and DEA estimates by presenting variety measure. In SFA with half-normal and truncated normal estimates, the average growth rate of TFP is 3.90% and 2.30%, respectively. Average growth rate of TFP is 2.95% from the DEA estimate. There is no record of technical regress when using SFA with half-normal distribution and SFA with truncated normal distribution, but DEA-based estimates show 10 years' technical regress throughout the study time. By all the methods of estimation, it is clear that the overall growth rate of TFP is positive and more than 2.30%, which supports the increase of rice production ability in Bangladesh [18]. SFA with half normal distribution shows that volatility of TFP growth is low. Maximum and minimum growth rate of TFP are found 4.67% and 3.1%, respectively. Maximum and minimum growth rates of TFP are 4.27% and 0.57%, respectively, in SFA with assumed truncated normal distribution and 27.53% and −18.73%, respectively, in DEA estimate. The SFA with half normal is measured higher than the TFP growth rate with less variation than that of DEA. This finding is supported by [19] and [20].

**Table 3:** Indirect Indicator of Plausibility of TFP Growth Results of Overall Rice Production.

	SFA (Half normal)	SFA (Truncated normal)	DEA
Average growth rate	3.90%	2.30%	2.95%
Maximum growth rate	4.67%	4.27%	27.53%
Minimum growth rate	3.1%	0.57%	−18.73%
Number of years recording negative TFP	0	0	10

**Figure 1:**
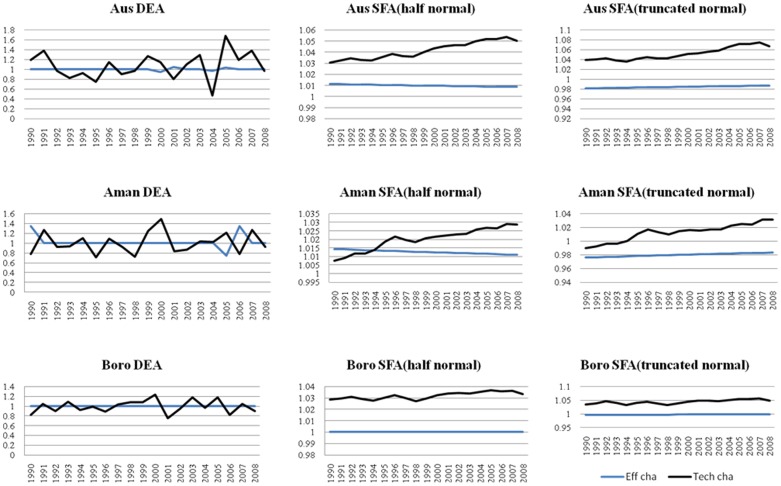
Efficiency change and Technical change of Aus, Aman and Boro in Bangladesh (1990–2008).

Figure 1 presents the efficiency change and technical change of rice production in Bangladesh by SFA and DEA. We observed the difference in the source of TFP change in the two methods, SFA and DEA. As expected, DEA-based series are much more volatile than that of SFA-based estimates. DEA-based estimates suggest technical regression and progression mixed in the rice production, but in contrast, half-normal and truncated normal SFA-based estimates indicate technical progression in all the years of production.

SFA with truncated normal shows that efficiency for Aus is negative but improving but constant from DEA (at 1.0) and SFA with half-normal (at 1.01) estimate. Efficiency change has fluctuation in certain years only for Aman in all methods of estimation. Fluctuation in efficiency change of Aman is observed in years 1990–1991 and 2004–2007 by DEA estimation. Efficiency change of Aman is always positive but decreasing in SFA with half-normal distribution, whereas SFA with truncated normal estimation is negative. Estimation based on DEA, SFA with half normal, and SFA with truncated normal shows that there is no efficiency change for Boro.

There are fluctuations in technical change for all types of rice by DEA estimation. Both methods of SFA (half normal and truncated normal) shows positive technical change. That is, technical change is always higher than that in the preceding year. For SFA estimation, technical progress is higher than the efficiency progress as shown in the graph; technical change shows upper line compared with efficiency change, except Aman in SFA with half normal. Technical change is less than the efficiency change in the first few years for Aman but otherwise in the following years. In this study, technical change, rather than the modest TFP growth, is the driving force in rice production in Bangladesh, whereas Nin and Yu [21] reported that efficiency change was the main force for TFP growth. Because of technical change, growth rate of TFP is more varied compared with efficiency change.

In Table 4, average growth rate of TFP of Aus is highest in case of SFA with half-normal distribution (5.2%) and DEA (7.6%), but SFA with truncated normal distribution shows that average growth rate of TFP is the highest for Boro rice. In case of DEA estimation of TFP, all the type of rice has found progression and regression. SFA with half normal and truncated normal shows that there is only progress in TFP for all types of rice except SFA with truncated normal distribution, which shows that there is regression and progression in TFP for Aman. Average growth rate of TFP is negative for Aman (−0.8%) in SFA with truncated normal distribution and Boro (−0.6%) in DEA. However, these negative growth rates of TFP suggested that the productivity growth is declining and the decline trend is a threat for economic development in Bangladesh [7].

**Table 4:** Growth Rate of TFP of Rice Production in Bangladesh by SFA and DEA.

Year	SFA (Half normal)			SFA (Truncated normal)			DEA		
	Aus	Aman	Boro	Aus	Aman	Boro	Aus	Aman	Boro
1990	4.2	2.2	2.9	2.0	−3.4	3.1	19.6	4.5	−18.3
1991	4.4	2.4	3.0	2.1	−3.1	3.4	38.7	27.0	4.6
1992	4.6	2.6	3.1	2.4	−2.7	4.2	−3.4	−7.6	−9.5
1993	4.4	2.6	2.9	2.0	−2.6	3.6	−16.9	−6.1	9.2
1994	4.3	2.8	2.8	1.8	−2.2	2.9	−7.0	9.9	−7.7
1995	4.6	3.2	3.0	2.4	−1.1	3.7	−25.2	−28.7	−1.6
1996	4.9	3.5	3.3	2.8	−0.4	4.1	14.7	9.5	−10.8
1997	4.7	3.3	3.0	2.6	−0.8	3.5	−9.1	−7.4	3.2
1998	4.6	3.1	2.8	2.6	−1.1	2.9	−3.4	−28.2	8.3
1999	5.0	3.4	3.0	3.1	−0.5	3.5	27.6	24.3	7.9
2000	5.4	3.4	3.3	3.6	−0.4	4.2	9.7	49.6	23.3
2001	5.5	3.5	3.4	3.7	−0.4	4.5	−15.4	−16.4	−24.4
2002	5.6	3.5	3.4	4	-0.2	4.5	9.9	−13.6	−5.6
2003	5.6	3.6	3.4	4.3	−0.1	4.4	29.2	3.6	17.6
2004	5.9	3.8	3.6	5.1	0.4	4.9	−54.2	2.0	−3.3
2005	6.1	3.9	3.7	5.7	0.7	5.3	74.0	−10.1	17.9
2006	6.1	3.8	3.6	5.7	0.7	5.2	19.6	4.5	−18.3
2007	6.3	4.0	3.7	6.1	1.4	5.3	38.7	27.0	4.6
2008	6.0	4.0	3.4	5.3	1.5	4.7	−3.4	−7.6	−9.1
Average	5.2	3.3	3.2	3.5	−0.8	4.1	7.6	1.9	−0.6

**Figure 2:**
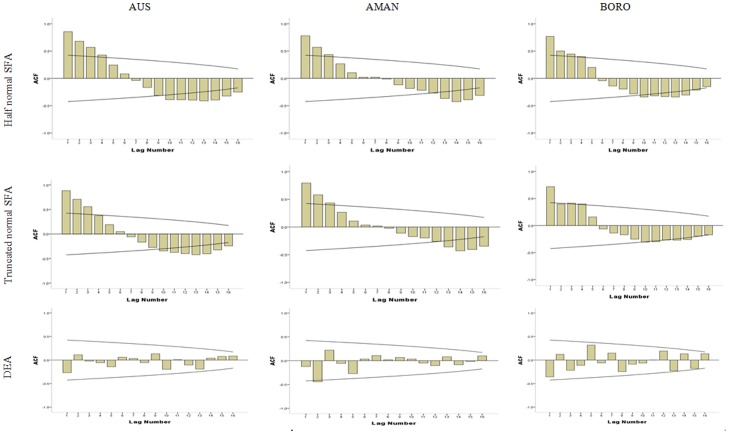
Correlogram of ACF for Identification of ARIMA Model.

**Figure 3:**
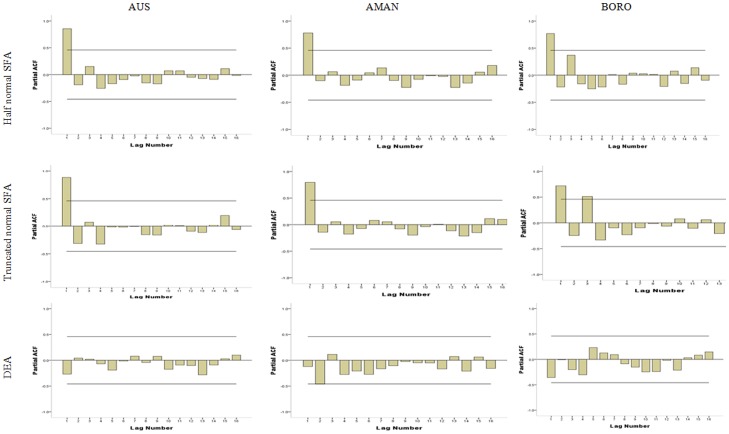
Correlogram of PACF for Identification of ARIMA Model.

Figure 2 and Figure 3 shows the plot of TFP for ACF and PACF. ACF shows the pattern of sine wave, whereas PACF shows a significant peak at lag 1 for all types of rice from SFA with half normal and truncated normal. These correlograms suggest using ARIMA (2, 0, 0) for forecasting models of TFP from SFA with half-normal and truncated normal distribution of Aus, Aman, and Boro. ACF and PACF from TFP of DEA estimations do not have significant peak for Aus and Boro, but for Aman, there is a significant peak at lag 2. TFP of Aman of DEA estimation can be forecasted with ARIMA (1,0,0).

Table 5 presents the estimated parameters for ARIMA model to forecast TFP. The estimated models for TFP from SFA with half-normal assumption are as follows:
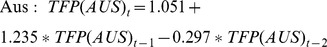
(11)

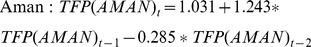
(12)

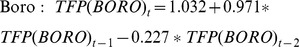
(13)The estimated models for TFP from SFA with truncated normal assumption are as follows:
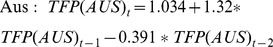
(14)

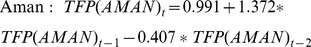
(15)

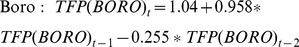
(16)And the estimated model for TFP of Aman from DEA is
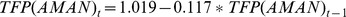
(17)In all the forecasting models of TFP from SFA, parameter of lag at 1 period has significant effect, and only Aman from SFA with truncated model has significance of both lag periods.

**Table 5:** Estimated Parameters for ARIMA Model to Forecast TFP.

	Aus		Aman		Boro	
	Parameter	estimate	Parameter	estimate	Parameter	estimate
Half normal SFA	AR1	1.235**	AR1	1.243**	AR1	0.971**
	AR2	−0.297	AR2	−0.285	AR2	−0.227
	Constant	1.051**	Constant	1.031**	Constant	1.032**
Truncated normal SFA	AR1	1.32**	AR1	1.372**	AR1	0.958**
	AR2	−0.391	AR2	−0.407*	AR2	−0.255
	Constant	1.034**	Constant	0.991**	Constant	1.04**
DEA	-	-	AR1	−0.117	-	-
	-	-	Constant	1.019**	-	-

**and * indicates significance level at 5 percent and 10 percent respectively.

## Conclusion

SFA and DEA is used to estimate the growth rate of TFP and efficiency of rice production in Bangladesh. We assumed two distributions (half normal and truncated normal) of inefficiency for translog stochastic frontier production model.

This paper presented some important findings for policy implication. Although the efficiency of Boro is followed by Aus and Aman, if we rank the average growth rate of TFP of rice production, SFA with half normal and DEA provide the same ranking (Aus>Aman>Boro). According to the performance of various types of rice, Aus is the major performer with an average TFP growth of 5.2% from SFA with half normal and 7.6% from DEA. Boro seems to be the weakest performer with negative TFP growth from DEA and 3.2% from SFA with half normal. We can conclude that Aus and Aman are improving their performance. Both SFA and DEA showed positive growth rate of TFP for Aus, indicating the improvement in technical efficiency. The TFP growth rate of Aman is negative in SFA with truncated normal estimation but positive in SFA with half normal and DEA estimation. Boro showed negative average growth rate of TFP in DEA estimation. Technical inefficiency is decreasing overtime, which is a great advantage for Bangladesh. Technical change is the driving force to improve TFP for rice production in Bangladesh. By increasing the efficiency level, the production of Aman and Aus can be increased. ARIMA (2,0,0) model is presented to forecast TFP of Aus, Aman, and Boro from SFA with half-normal and truncated normal assumption, and ARIMA (1,0,0) model is used to forecast the TFP of Aman from DEA estimation. TFP of Aus and Boro from DEA estimation cannot be forecasted by ARIMA.
